# Utility of local health registers in measuring perinatal mortality: A case study in rural Indonesia

**DOI:** 10.1186/1471-2393-11-20

**Published:** 2011-03-17

**Authors:** Leona Burke, Dwi Linna Suswardany, Keryl Michener, Setiawaty Mazurki, Timothy Adair, Catur Elmiyati, Chalapati Rao

**Affiliations:** 1 School of Population Health, University of Queensland, 288, Herston Road, Herston QLD 4006, Australia; 2 District Health Office, Pekalongan District, Central Java, Indonesia

## Abstract

**Background:**

Perinatal mortality is an important indicator of obstetric and newborn care services. Although the vast majority of global perinatal mortality is estimated to occur in developing countries, there is a critical paucity of reliable data at the local level to inform health policy, plan health care services, and monitor their impact. This paper explores the utility of information from village health registers to measure perinatal mortality at the sub district level in a rural area of Indonesia.

**Methods:**

A retrospective pregnancy cohort for 2007 was constructed by triangulating data from antenatal care, birth, and newborn care registers in a sample of villages in three rural sub districts in Central Java, Indonesia. For each pregnancy, birth outcome and first week survival were traced and recorded from the different registers, as available. Additional local death records were consulted to verify perinatal mortality, or identify deaths not recorded in the health registers. Analyses were performed to assess data quality from registers, and measure perinatal mortality rates. Qualitative research was conducted to explore knowledge and practices of village midwives in register maintenance and reporting of perinatal mortality.

**Results:**

Field activities were conducted in 23 villages, covering a total of 1759 deliveries that occurred in 2007. Perinatal mortality outcomes were 23 stillbirths and 15 early neonatal deaths, resulting in a perinatal mortality rate of 21.6 per 1000 live births in 2007. Stillbirth rates for the study population were about four times the rates reported in the routine Maternal and Child Health program information system. Inadequate awareness and supervision, and alternate workload were cited by local midwives as factors resulting in inconsistent data reporting.

**Conclusions:**

Local maternal and child health registers are a useful source of information on perinatal mortality in rural Indonesia. Suitable training, supervision, and quality control, in conjunction with computerisation to strengthen register maintenance can provide routine local area measures of perinatal mortality for health policy, and monitoring of newborn care interventions. Similar efforts are required to strengthen routine health data in all developing countries, to guide planned progress towards reduction in the local, national and international burden from perinatal mortality.

## Background

Reliable measures of stillbirths and early neonatal deaths (i.e perinatal mortality) are necessary for planning and evaluation of prenatal, obstetric, and newborn care services[1]. It has been estimated that perinatal mortality accounts for about 7% of the total global burden of disease[2]. Of 133 million births worldwide in 2004, 5.9 million were estimated to have died during the perinatal period, and the vast majority of these deaths occurred in developing countries, including about 97,000 perinatal deaths in Indonesia[3].

Measures of perinatal mortality can be derived using data from vital statistics, routine health services data, or sample surveys[4]. Of these, vital statistics are the optimal data source for perinatal mortality. However, in developing countries, incomplete registration of births and deaths results in inaccurate vital statistics [5]. Also, available or reported data from local or national health services information systems are potentially affected by incomplete coverage as well as information bias[6]. Hence, the most commonly utilised data are from surveys, which form the main source for estimates of perinatal mortality in developing countries such as Indonesia, given the absence or weakness of data from routine sources [7].

Sample surveys estimate perinatal mortality based on retrospective complete birth histories from women of reproductive age. Recent surveys indicate that perinatal mortality in Indonesia has remained stagnant at around 25 per 1000 live births over the past decade[8]. However, sample surveys such as the Indonesian Demographic and Health Survey (IDHS) generate mortality measures only at the regional or provincial level in Indonesia, and may mask differentials at the district/sub district level. Moreover, implementation of surveys require additional human, financial and technical resources, and the data are prone to recall and information bias, particularly regarding stillbirths[4].

With the shift to district level decentralised health service provision in Indonesia,[9] there is a need for regular and accurate local measures of perinatal mortality at the district and even sub-district level, for planned improvements in maternal and newborn care services. Given the challenges in operating efficient vital registration, and the limitations of sample surveys, robust maternal and child health services data offer much promise as a source for measuring perinatal mortality at the local level.

This paper describes research undertaken in a rural district in Indonesia, to study the utility of local health registers on pregnancies, births and deaths in measuring perinatal mortality at the local level. Information from health service registers was triangulated with information from a range of other sources that record deaths, to estimate perinatal mortality rates at the sub district level in Indonesia. The study findings were compared with local vital statistics, as well as with the data on pregnancies and perinatal deaths reported for the study population by the Maternal and Child Health Program. This research demonstrates the feasibility and potential in using locally available health services data to improve perinatal mortality measurement in Indonesia.

## Methods

### Study population

The study was conducted in Pekalongan district, Central Java Province, Indonesia. Pekalongan was selected as three of its subdistricts (Kajen 1, Kedungwuni 1, and Wonokerto) were involved in a pilot field project being conducted to strengthen mortality registration and vital statistics systems in Indonesia[10]. Initial findings from the mortality registration project identified under-reporting of perinatal events in 2006-2007. Also, annual reports for this study population by the Maternal and Child Health (MCH) Program are implausible, with no stillbirths being reported from two out of the three study subdistricts [11]. Hence, this study was designed to explore the potential to identify perinatal deaths in local health service registers for 2007, as a data source to strengthen perinatal mortality registration in the main project.

The three sub districts include a total of 34 villages. Each sub district is served by a government health centre (*puskesmas*), which deploys a government midwife to each village within the sub district to provide maternal and child health services. The field research team comprised a group of four research staff from the School of Population Health, University of Queensland, working in collaboration with local government health personnel in Pekalongan district.

### Data sources

As part of routine maternal and child health services, the government employed midwives in each village maintain separate registers to record their activities as follows:

a) pregnancy registration and antenatal care services (*ibu kohort *register),

b) birthing support services including record of birth outcomes (*partus *register), and

c) infant health services including immunization and growth monitoring (*bayi kohort *registers)

In addition to the registers, the midwives maintain informal notes in a handbook, which they later transcribe into the registers on a periodic basis. The midwives also submit a monthly statistical report to the District Maternal and Child Health Program, in which they provide data on the number antenatal cases attended, along with data on deliveries, stillbirths, and infant deaths. These reports are compiled at the District Health Office into annual MCH Program reports. The village midwives also maintain a separate death register for each village, which is in addition to several other data sources on deaths, including the village administration death register, which is the official local source for vital statistics. Table 1 shows the list of data sources used in this study.

**Table 1:** Data sources for measuring perinatal mortality in study population

Pregnancies and perinatal deaths (health registers)	Additional sources for perinatal deaths
Pregnancy (ibu kohort) register	Village midwife death register
Delivery (partus) register	Midwife health register follow up
Infant (bayi) register	Village administration death register*
	Health centre death register
	Health centre verbal autopsy questionnaires

* Official source for local vital statistics

### Data collection

At first, the study team closely examined the structure and content of the three registers, and developed a comprehensive data collection format to record key variables necessary to match, track and record perinatal survival in the pregnancy cohort from each village. In each sub district, data collection was preceded by a half-day briefing attended by all participating village midwives, which covered the objectives of the study and the core data collection methods.

The field research was conducted in February-April 2009. Fieldwork was conducted in each sub-district over a period of two weeks. As an initial step in each village, the research team (in collaboration with the village midwife) collated all records to develop a complete list of deliveries that occurred in 2007. The key variables that were used to match records across registers were the name of the mother & father (and child if available), date of birth, and where applicable, the date of death, and age at death. Village midwives were consulted to verify unmatched pregnancy and birth records, leading either to reconciliation since they actually pertained to a single event or to their identification as truly unique pregnancies in the perinatal cohort. For each pregnancy, the date of delivery, duration of gestation and birth outcome was recorded based on data available from the three registers. We applied the stillbirth definition prescribed by the World Health Organization for reporting perinatal mortality for international comparison (i.e fetal death after 28 weeks gestation) [12]; to enable comparison with data from the maternal and child health program, and the Demography and Health Surveys.

For some pregnancies, the outcome was not recorded in the pregnancy register or the delivery register, but was identified as a live birth since the child received immunization as recorded in the infant register. In other instances for which no register entry was available indicating the outcome, midwives were asked to consult their informal notes, or local traditional birth attendants who had assisted with the concerned delivery, and complete the record. This component of data collection was termed 'Midwife follow up of health registers'.

All live births were followed up in the infant register to trace and record survival or death in the first week of life. Survival was confirmed by an entry for immunization at four weeks age. Missing entries were investigated as per the register follow up method described above, and in case of death, the exact age at death in days was recorded in the study dataset. In addition, local death registers maintained by the village administration or the health centre were consulted to identify deaths in the first week as well as stillbirths, and these were used to verify entries in registers, or complete records, as necessary.

Following collation of all records with suitable data linkage, a comprehensive electronic database was created to enter the records pertaining to each pregnancy, its outcome and if a live birth, its survival through the first week, for each study village. Key variables captured for each record from different data sources include identification variables, date of birth, birth outcome, length of gestation, and date of death, among others. The complete set of variables collected for each record from each data source is listed in Additional File 1. Data were aggregated across villages in each sub district, and across the entire sample, for descriptive statistical analyses.

Ethics clearance for the study was obtained from the Institutional Review Board at the University of Queensland, and from the Ethical Review Committee at the National Institute of Health Research and Development, Ministry of Health, Indonesia. Local permission and oversight of the field study was provided by the District Health Office in Pekalongan.

### Descriptive analysis

The data were first scrutinised to assess the completeness of information recorded in key fields in each health register, such as birth outcome, date of birth, birth weight, and duration of gestation. The completeness of pregnancy registration in each register was evaluated as a percentage of the entire cohort. Identified perinatal events were triangulated with data from other sources of information on vital events, to develop a comprehensive list of still births, live births and early neonatal deaths, to derive measures of perinatal mortality rates for each sub district. Finally, data on perinatal deaths (i.e stillbirths at > 28 weeks gestation plus deaths in the first week of life) were used to calculate perinatal mortality rates for the study population.

### Qualitative methods

The study included the use of qualitative research methods to study the experiences of midwives in recording and reporting perinatal deaths. In each sub district, midwives from all villages included in the study attended a focus group discussion held at the health centre, lasting about 90 minutes each. Informed consent was obtained from each participant, and discussions were conducted in the national language (*Bahasa Indonesia*). Electronic audio tapes were then transcribed and translated into English, providing a verbatim note of what was said. The themes explored in the discussions included enquiry into their work practices, their interaction with Traditional Birth Attendants, and their opinions on the strengths and weaknesses of existing recording and reporting systems. Coding or indexing was done for specific themes and probes used to facilitate the discussion, and provide a framework for thematic analysis. In this paper, we only report specific issues that provide insight into the errors in register maintenance, and the reasons for such error.

## Results

Overall, field activities were conducted in 23 out of 34 villages. Reasons for non-participation included recent appointment of new personnel not fully aware of recording practices and maintenance of registers in 2007 (4 villages), non-availability of registers for 2007 (2 villages), absence of midwives during the period of contact due to personal reasons (3 villages), and in the remaining 2 villages, midwives were involved with other health program activities, and could not participate in the research.

### Completeness and quality of information from registers

Overall, the scrutiny of health registers indicated a uniformly poor quality of data recording of birth outcome and date of birth in all villages included in the study. Table 2 shows the measures of completeness of some of the key fields that are required to assess perinatal survival from the registers. Apart from incomplete recording, there were also problems with the design of the registers. For instance, the pregnancy register did not contain a field to record the date of the last menstrual period. Further enquiry revealed that this date was only recorded on the individual antenatal care card maintained for each registered pregnant woman. Also, there was no specific field in the pregnancy register to record the length of gestation at delivery, which is essential to apply the criteria for defining stillbirths. On detailed scrutiny of the registers, the midwives clarified to us that they followed an informal practice of noting the estimated duration of gestation in months within the column for recording the date of each antenatal visit, in order to keep track of this important variable. However, we noted that there was variation in the implementation of this practice across villages. The delivery register did contain a column for recording the length of gestation at birth, but this was completed only in the registers maintained by midwives in the study villages from Wonokerto sub district. Finally, while the birth weight was recorded in the delivery register, in some villages all weights were recorded as 2500 grams, indicating that no serious attention was being paid to accuracy of the record. It is also noted here that there were several other information fields e.g. name of mother, age of mother, address, which were either not recorded or recorded partially, or even recorded differently in different registers, which created major obstacles in the ensuing matching task conducted to develop the pregnancy cohort.

**Table 2:** Completeness of key data fields to assess birth outcomes from health registers maintained by village midwives serving the study population, Pekalongan, 2007

Health register	Total pregnancies	Register fields			
		
		Birth outcome	Birth date	Length of gestation	Birth weight
*Ibu kohort *(Antenatal)	1140	244 (21%)	538 (47%)	No field in register*	No field in register
*Partus *(Birth)	1225	505 (41%)	800 (65%)	367 (30%)	1136 (93%)
*Bayi *(Infant health)	1262	Not applicable	1223 (97%)	Not applicable	No field in register

*The ibu kohort register did not have a field for recording the date of the last menstrual period, it was only recorded on the individual antenatal card issued to each registered pregnant woman

### Completeness of pregnancy registration in health registers

**Figure 1:**
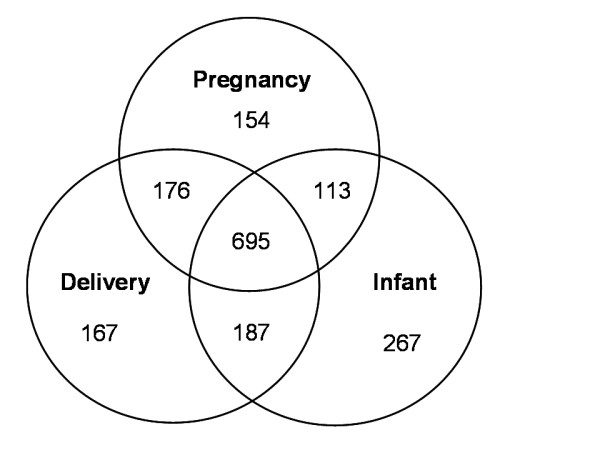
**Distribution of pregnancies identified across local health registers in the study sample of villages in Pekalongan, 2007**.

A total of 1759 pregnancies that resulted in delivery were identified after data linkage across the three health registers. The distribution of pregnancies across the registers is illustrated in Figure 1. Data linkage indicated that while each of the three registers had recorded between 65-73% of the total pregnancies, only 695 (40%) were recorded in all three registers. Similar analyses conducted at the sub district level identified variations in the proportion of births common to all three registers with Kajen demonstrating the highest percentage (48%); followed by Kedungwuni (38%) and Wonokerto the least (33%).

### Perinatal deaths and mortality rates

The recording of perinatal deaths can be expected to vary across registers. For instance, pregnancy registers should at least record all stillbirths, delivery registers could be expected to record all stillbirths and at least early neonatal deaths within the first few hours of life; and the infant registers will not include records of stillbirths, but could be expected to record all early neonatal deaths. As described in the Methods, we also pursued a range of additional sources to identify perinatal deaths that could have occurred in the study population during the reference period. Again, appropriate matching was conducted to develop a final unique list of perinatal deaths.

Table 3 shows the distribution of stillbirths and early neonatal deaths captured in each of the health registers, and from all other sources used in the study. In summary, none of the data sources for deaths was complete. Overall, the midwife registers were found to have recorded 74% of perinatal deaths identified in the study population, including 19 out of 23 stillbirths (83%), and 9 of the 15 early neonatal deaths (60%). In contrast, the village administration death register (official source for local vital statistics) recorded only 15 out of the 38 perinatal deaths; about 40%.

**Table 3:** Distribution of perinatal deaths identified in study populationfrom different data sources

Data source	Stillbirths	Early Neonatal Deaths	Total Perinatal Deaths
***Health registers***			
Pregnancy register	14	3	17
Partus register	13	5	18
Baby register	0	2	2
**Total deaths after data linkage**	**19**	**9**	**28**

***Additional sources***			
Midwife village register of deaths	11	8	19
Midwife health register follow up	1	5	6
Administration village death register	6	9	15
Health centre register of deaths	16	5	21
Health centre verbal autopsy questionnaires	11	9	20

**Total deaths all sources after data linkage**	**23**	**15**	**38**

Table 4 compares the study findings on births and deaths from linked registers with the reported MCH program data for each sub district. The comparison shows serious under reporting in the MCH program data. For the 23 villages comprising our study population, we measured a stillbirth rate of 13.5 per 1000 total births, which was over four times the stillbirth rate (8/2228; or 3.5 per 1000 births) reported to the district health department for all 34 villages located in the three sub district. In terms of perinatal mortality rates, our measures for Kajen 1 and Kedungwuni 1 are marginally higher than the point estimate of perinatal mortality rate from the Indonesian Demographic Health Survey for Central Java during 2003-2006, which was 20.3 per 1000 total births (95% CI 8.7 - 31.8)[13].

**Table 4:** Comparison between final study data and MCH program data for the study sub districts in Pekalongan, 2007

Sub district	Data from study population					Data reported to MCH Program			
	
	Study villages	Total births	Still births	Early neonatal deaths	Perinatal mortality rate*	Total villages	Total births	Still births	Infant deaths
**Kajen 1**	**8**	**625**	**10**	**5**	**24**	**11**	**625**	**0**	**1**
**Kedungwuni 1**	**7**	**468**	**7**	**4**	**23.5**	**12**	**883**	**8**	**7**
**Wonokerto**	**8**	**666**	**6**	**6**	**18.1**	**11**	**720**	**0**	**0**
**Total**	**23**	**1759**	**23**	**15**	**21.6**	**34**	**2228**	**8**	**8**

* per 1000 live births

#### Reasons for low routine data quality

Qualitative research identified that the majority of women in Pekalongan deliver at home, attended by the trained health centre midwife. Several health centres have delivery rooms, but these are not used. Some women prefer a Traditional Birth Attendant, but the trained midwife is also present. In case of complications, the case is shifted to the district hospital. A small number of women attend private health facilities in the town or capital cities. In all such cases of delivery in health facilities, the details of delivery are recorded on discharge cards, and these details are entered in the local village delivery register. The above variations in birthing circumstances had an obvious influence on the manner in which data were recorded in the registers. In addition, the qualitative research identified several other reasons for the inconsistent register maintenance. These could be largely described in the following categories:

a) High workload on midwives, with little time for routine documentation. This is coupled with a lack of awareness about the importance of data accuracy for health monitoring and health services planning. This causes the midwives to maintain informal notes in handbooks (rather than the bulky registers), which they later use to update the main registers in the immediate period prior to submitting monthly returns. This results in losses during transcription due to use of shorthand, misspellings, illegibility, inaccurate memory, and other oversight while recording data in handbooks.

b) Individual variations and inconsistencies in accessing local health services during pregnancy, childbirth and post partum. This leads to duplication of records or losses to follow up, hence complicating records maintenance which compromises data accuracy.

c) Absence of any local supervision in an environment where data in the monthly report is used to measure accomplishment of service targets set for local staff, which leads to inconsistencies between data in the registers and the data in the summary monthly reports.

d) Inadequate collaboration and data sharing with local Traditional Birth Attendants, who also provide birthing and post natal services to the community.

e) Lack of coordination with other responsible stakeholders within the community who are also responsible for gathering/reporting information on vital events e.g. village administration; community volunteers (*rukun tetanga*/*rukun warga*).This results in discrepancies between data recorded in the different data sources maintained at the local level.

## Discussion

This study set out to explore the potential to derive local measures of perinatal mortality using routine data sources at the village and sub district level in Indonesia. The research identified that despite several limitations in the maintenance of records and in current reporting systems, there is adequate information available at the local level for this purpose. Also, although not described here, the qualitative research conducted in association with this study confirms the changing and complementary relationship between the government, private and traditional antenatal and birth assistance services in rural Indonesia, at least in rural areas of Java. In such a complex environment, it is necessary to have robust local measures of birth services and outcomes, along with other useful information to assist health monitoring and provision of maternal and child services, such as the incidence of prematurity and low birth weight. Of course, such goals could be realised only if the limitations in the routine data collection systems identified by this study are addressed through appropriate interventions to strengthen the local maternal and child health information systems, and improved data sharing mechanisms with the non-government sector as well as with the local civil registration of stillbirths, births, and deaths. Improved identification of perinatal events could also lead to further investigation into the distribution of likely maternal and foetal causes of stillbirths and early neonatal deaths, through the use of Indonesian adaptation of verbal autopsy methods, as developed and described elsewhere[10].

Our approach in exploring the potential to measure perinatal mortality using information from local health registers is novel, but is similar to investigations in China using detailed family planning program records, [14] or in the Netherlands, using electronic registers maintained by different stakeholders [15]. Nevertheless, the findings are clearly indicative of the problems with official statistics, as observed in other countries [16,17].

Our estimates of perinatal mortality for each sub district may have been biased by the exclusion of certain villages from the study sample, reasons for which have been provided in the Results. Also, there were significant problems in the matching of events across registers, due to variations in recorded names of the mother, the absence of critical variables in different registers such as the date of delivery/birth, and the length of gestation or age at death. Another important limitation was the availability of information on duration of gestation at pregnancy outcome, which hampered accurate determination of stillbirths. We identified a total of 50 cases of fetal death, but we were able to confirm gestation ≥ 7 months (≈ 28 weeks) in only 23 cases. Of the remaining 27 cases, we were able to confirm gestation ≤ 6 months in only 17 cases, while in the remaining 10 cases, the exact gestational age at fetal death could not be determined. In our analysis, we chose to classify these 10 cases as miscarriages. This could have resulted in under estimation of stillbirths, as a result of improper application of the 28 week definition.

Further, the absence of reliable information on birth weight precluded the possibility of applying more sensitive criteria such as the 1000 g threshold for stillbirths. It has been observed that on average, fetal weight at 28 weeks exceeds 1000 g [18]. Hence, if accurate data on birth weight were available, the use of the 1000 g threshold would have probably increased the stillbirth, and therefore, perinatal mortality rates. We believe therefore that our measures of perinatal mortality are more likely to be the lower limit of the true perinatal mortality rates in these communities.

There is also potential for bias from misclassification between stillbirths and early neonatal deaths, however, the perinatal mortality rate adequately accounts for such misclassification. At the same time, it has been recommended to present data on stillbirths and early neonatal deaths separately, given their distinct epidemiological differences and health service implications[19]. Therefore, we chose to present our detailed findings in Tables 3 and 4, for any future comparative analyses. Finally, for all live births, there could have been losses to follow up or misclassification of survival beyond one week. We explored a range of data sources on early neonatal deaths (see Table 4) and linked them to the pregnancies and deliveries, thereby minimizing missed deaths from losses to follow up or misclassification. While a detailed prospective study that closely monitors birth outcomes and survival in a pregnancy cohort would be the ideal study design to avoid losses to follow up and misclassification, such studies are resource-intensive and time-consuming. We believe that our retrospective study design to match all pregnancies with birth outcomes and early neonatal survival is an adequate alternative for the measurement of perinatal mortality from routine health registers.

A recent evaluation of community based interventions to improve the quality of newborn care and reduce perinatal mortality identified that the interventions were partially successful, resulting in significant reductions in stillbirth rates, but without any impact on early neonatal deaths [20]. This suggests that reductions in perinatal mortality would require community based interventions in combination with other clinical care intervention packages that would require more advanced facilities, technology, and skilled human resources. However, research in other settings has demonstrated that community based or primary health care interventions do have value in reducing perinatal mortality[21,22]. It would be safe to conclude that accurate measures of perinatal mortality are necessary for monitoring and planning of interventions to improve birth outcomes.

Also, declines in under-five and infant mortality rates lead to increasing proportionate mortality during the neonatal period, most of which occurs within the first week of life[23,24]. Such declines in under-five and infant mortality are currently being experienced in many developing countries progressing towards United Nations Millennium Development Goal 4, including Indonesia[25]. Therefore, in order to achieve further and continued reductions in under-five mortality, it is necessary to measure and address the burden from perinatal mortality.

## Conclusions

In the current environment, there appears to be a growing need and demand for local measures to assist planned health care delivery[26]. Vital statistics systems in Indonesia are still under development, and have not yet realised their utility as a data source for measuring local mortality[27]. Therefore, the findings from this study demonstrate the potential to measure perinatal mortality at the local level, using available data from maternal and child health program registers, in combination with local vital registration systems. Our findings call for urgent attention to strengthening the performance of the MCH Program data reporting system, through appropriate guidelines on the maintenance of local registers, and on accurate reporting of MCH services data by village midwives. These data should also be linked with the official vital statistics systems, as mandated in recent Indonesian legislation and guidelines on this subject[28,29]. On a periodic basis, research studies such as the one described in this article could be conducted to validate local vital statistics or MCH program data. Such improved practices will help generate routine and timely measures of perinatal deaths and their causes for planned improvements in newborn care.

## Competing interests

The authors declare that they have no competing interests.

## Authors' contributions

LB participated in the data collection, analysis, and drafted the initial version of the manuscript. DS, KM and SM participated in data collection and analysis, and contributed to individual sections of methods, results and discussion sections of the manuscript. CR, TA and CE conceptualised the research study, provided guidance on the field data collection and analysis, and provided critical appraisal and revisions to the manuscript prior to submission. All authors read and approved the final version of the manuscript.

## Funding

The field research conducted by LB, DS, KM and SW was supported by a research grant from the Australian Agency for International Development (ROU 13652), which included support to the University of Queensland staff (TA and CR). CE is a government employee of the District Health Office, Pekalongan, Indonesia. The funding body did not have any role in the study design, data analysis, or manuscript preparation and submission.

## Pre-publication history

The pre-publication history for this paper can be accessed here:

http://www.biomedcentral.com/1471-2393/11/20/prepub

## Supplementary Material

Additional File 1 **Table S1: Principle variables collected from local health registers and additional sources**. Click here for file
