# Supplementary material for: Utility of local health registers in measuring perinatal mortality: A case study in rural Indonesia
Source: BMC Pregnancy Childbirth. 2011 Mar 17;11:20. doi: 10.1186/1471-2393-11-20 (PMC3068126; doi:10.1186/1471-2393-11-20)
Supplement: Additional File 1 — Table S1: Principle variables collected from local health registers and additional sources. [file 1471-2393-11-20-S1.DOC]

Table S1: Principle variables collected from local health registers and additional sources

| **Health Registers** | | **Additional Sources** | |
| --- | --- | --- | --- |
| *Source* | *Data collected* | *Source* | *Data collected* |
| Pregnancy register | - Name of mother - Name of father - Birth outcome - Date of birth of baby - Comments | Midwife health register follow up questionnaires | - Birth outcome - Survival of child - Date of birth - Date of death - Reason for missing information - Comments |
| Partus register | - Name of mother - Name of father - Month birth was reported in register - Length of gestation - Birth outcome - Date of birth of baby - Comments | Village administration death register | - Name of parent - Name of baby - Date of birth - Date of death - Cause of death - Age at death - Comments |
| Baby register | - Name of baby - Date of birth - Name of mother - Name of father Immunisations dates - Date of death - Cause of death - Comments | Health centre verbal autopsies | - Name of baby - Village - Date of birth - Age at death - Date of death - Cause of death - Comments |
| **Death Registers** | | Traditional birth attendants | - Name of mother - Name of father - Name of baby - Date of birth - Survival of child - Date of death - Comments |
| Village midwife death register s | - Name of baby - Date of birth - Date of death - Cause of death - Comments |
| Health centre death register | - Name of baby - Date of birth - Date of death - Age at death - Cause of death - Comments | Midwife Follow Up | - Personal knowledge of child - Information gathered from other health centre documentation. |
